# Bipolar Disorder: From Pathophysiology to Treatment

**DOI:** 10.14789/jmj.JMJ21-0026-R

**Published:** 2021-12-17

**Authors:** TADAFUMI KATO

**Affiliations:** 1 Department of Psychiatry and Behavioral Science, Juntendo University, Faculty of Medicine, Tokyo, Japan; 1 Department of Psychiatry and Behavioral Science, Juntendo University, Faculty of Medicine, Tokyo, Japan

**Keywords:** depression, mania, lithium, mood stabilizers, anticonvulsants

## Abstract

Bipolar disorder is a mental disorder that involves a manic or hypomanic state and a depressive state, and was once called manic-depressive disorder and was considered one of the two major mental disorders along with schizophrenia. Major depressive disorder, on the other hand, is a disorder in which only depressive states occur, and the two are sometimes referred to together as “mood disorders. This review will introduce the pathophysiology, diagnosis, epidemiology, and treatment of bipolar disorder, focusing on the current situation in Japan.

## Introduction

Bipolar disorder is a psychiatric disorder characterized by recurrent manic or hypomanic and depressive states, and was formerly called “manic- depressive illness” and was considered one of two major psychiatric disorders along with schizophrenia. Major depressive disorder is a disorder in which only the depressed state occurs. The two are sometimes referred to together as “mood disorders”, though DSM-5 discarded this category because bipolar disorder also shares some features with schizophrenia. One manic episode is sufficient for the diagnosis of bipolar I disorder, whereas at least one hypomanic episode and one major depressive episode are necessary for the diagnosis of bipolar II disorder.

In this review, pathophysiology, diagnosis, epidemiology and treatment of bipolar disorder are summarized, and the current situation in Japan is also introduced.

## Symptoms

Bipolar disorder is characterized by two contrasting mental states, that is, manic or hypomanic episodes and depressive episodes. Between these episodes, patients experience euthymic, remission states.

## Manic state

In the manic state of bipolar I disorder, a patient moves around without sleeping and continues to talk without rest, and because of this, the family is exhausted. While being active, the patient cannot focus on one thing and cannot work efficiently. In addition, it is common for a manic patient to make expensive purchases, make large amounts of debt, or cause legal problems. In many cases, they lose their social credibility by making unreasonable efforts that are likely to fail. It may develop into a grandiose delusion such as having supernatural powers.

In a manic state, the patient is often unaware of his or her changes and feels refreshed and in better shape than usual, so even if he or she is having trouble, often the patient does not have an awareness that he or she is in trouble or annoys others.

## Hypomanic state

On the other hand, in the hypomanic state of bipolar II disorder, the patient is energetic as if he or she has changed from the usual, becomes active in human relations, moves around without hesitation even after a short period of sleep, and looks clearly “high” to those around him or her compared to the usual. However, they do not cause trouble to the people around them as in a manic state.

## Depressive state

While the manic state is the most troubling symptom for the family, the depressive state is the most painful for the patient. The two core symptoms of depression are “depressed mood,” which is a feeling of inexpressible annoyance that lasts all day, every day, and “loss of interest and pleasure,” which is a loss of interest in everything and the inability to feel happy or joyful in anything. The presence of at least one of these two symptoms is considered necessary for diagnosis.

Including these two essential symptoms, depression is defined as the daily occurrence of five or more of the various symptoms of depression for two weeks or more, such as sleep disturbance, decreased or increased appetite (and weight loss or gain), fatigue, psychomotor retardation (slowing down of movements), guilty feeling, loss of concentration, and suicidal thoughts.

## Clinical course

In bipolar disorder, there is often an interval of about five years between the first episode (depression or mania) and the next. During the remission state, when mania and depression have subsided, there are no symptoms, but if preventive therapy is not used, relapse will occur in most cases. If left untreated, the inter-episode interval gradually shortens over time^1)^, and if the disease shifts to rapid cycling (four or more episodes per year), it becomes difficult to respond to pharmacotherapy.

Comparing the duration of the manic state and depressive state, the duration of the depressive state is longer, and the patient is often unaware of the manic or hypomanic state, so many patients visit the clinic in a depressed state. However, in many cases, patients do not tell their doctors about their previous manic or hypomanic states at the time of their visit, in which case they are diagnosed with major depressive disorder and do not receive appropriate treatment.

Bipolar disorder is a serious illness that can be life-threatening in its depressive state due to suicide and socially life-threatening in its manic state due to the consequences of its behavior. On the other hand, bipolar patients are also known to be at increased risk for cardiovascular disease, and cardiovascular disease is the leading cause of death in bipolar patients^2)^.

## Epidemiology

Worldwide, bipolar I disorder is considered to affect around 1% of the population, and 2-3% if both bipolar I and II are included^1)^.

On the other hand, in Japan, epidemiological studies large enough to estimate the prevalence have not been conducted, and the lifetime prevalence of bipolar I and II patients combined is reported to be about 0.16 to 0.6%^3-5)^, which is lower than in Europe and the United States. In the United States, the prevalence of bipolar disorder is lower in Asians compared with other ethnicities, suggesting that different genetic backgrounds may underlie this difference^5)^.

It is reported that 16% of patients who are being treated for the depressive state are bipolar disorder^6)^, which is a higher percentage than the difference in lifetime prevalence (lifetime prevalence of depression is about 15% worldwide and about 7% in Japan). This may be because, unlike depression, in which about a half of the patients experience only a single episode for a lifetime, bipolar disorder is characterized by multiple recurrent manic and depressive episodes.

## Diagnosis

Bipolar disorder is classified into two categories based on the degree of mood elevation. The manic state is so severe that it interferes with family and work and requires hospitalization; On the other hand, the hypomanic state is a state of mood elevation in which the person is clearly different from the person as usual but is not so severe to require hospitalization.

Bipolar disorder with a manic state is called bipolar I disorder. Even in cases where the only manic state is observed, a depressive state often appears in the course of the disease, and the diagnosis of bipolar I disorder is made even when no depressive state is seen^7)^.

On the other hand, bipolar disorder with both hypomanic state and depressive state is called bipolar II disorder.

If left untreated, bipolar I disorder can lead to multiple cycles of manic and depressive states, during which the foundation of one’s life, such as relationships, social trust, work and family, is greatly damaged. However, treatment and coping strategies for bipolar disorder have been formulated, and in many cases, it is possible to control the illness and lead a social life.

When a depressive patient visits a clinic, the criteria for a depressive episode are checked after ruling out depression due to general medical diseases or depression due to substances or drugs. In addition, the presence or absence of a history of manic or hypomanic states should be checked. To diagnose major depressive disorder, it is necessary to confirm the absence of a history of manic or hypomanic states. However, it is not easy to accurately diagnose past hypomania, and the diagnosis of bipolar II disorder, in particular, tends to vary among doctors^8)^. The depressive state of bipolar disorder is characterized by a family history of bipolar disorder, young age of onset (less than 25 years old), and psychotic symptoms (e.g., auditory hallucinations, delusions)^9)^, but these alone cannot be used to diagnose bipolar disorder, and there is still no test that can help differentiate between the two. In Japan, near-infrared spectroscopy (optical topography) is covered by insurance as an aid in the differential diagnosis of depression, but there is little evidence that this method is actually useful for differential diagnosis, and worldwide, this method is not considered to be useful for differentiating depression from bipolar disorder^10)^. Although many other biomarkers for differentiating depression and bipolar disorder have been studied, none have been confirmed in multiple studies and have established diagnostic significance^11)^.

Both manic and depressive states may present with psychotic states such as delusions, auditory hallucinations, or catatonic states such as stupor.

When manic states first appear, it may be difficult to distinguish them from schizophrenia if psychotic symptoms are in the foreground. In addition, patients who initially present with a short-term psychotic disorder may subsequently develop bipolar disorder.

## Treatment

### Treatment goals

In the treatment of the major depressive disorder, the goal is to cure the depressive state, and in most cases, the treatment is terminated after about one year of recovery. On the other hand, in the case of bipolar disorder, the manic and depressive states will eventually be cured even if left untreated, but the manic and depressive states recur in most cases, so the goal of treatment is to prevent these episodes, and the key to treatment is how to prevent the manic and depressive states after they are cured. If treatment is stopped after the manic state is ameliorated, relapse will occur repeatedly, resulting in significant social damage and may also cause cognitive dysfunction^12)^.

It is not easy to continue medication for a lifetime in a state of remission when symptoms have subsided, and a combination of pharmacotherapy and psychosocial intervention that promotes acceptance of the disease is necessary. Patients go through various stages before accepting the disease, such as doubting the doctor’s diagnosis, doctor shopping searching for another, better diagnosis, self-stigma, and anxiety about relapse. Therefore, it is important to monitor how the patient perceives the disease and to provide psychotherapeutic treatment according to the stage of the disease.

## Pharmacotherapy

Medications other than antipsychotics used in the treatment of bipolar disorder are called mood stabilizers. The mood stabilizers used in Japan include lithium and three antiepileptic drugs: lamotrigine, valproate, and carbamazepine^13)^.

Atypical antipsychotics such as quetiapine, olanzapine, and aripiprazole are also used. In addition to these drugs, the atypical antipsychotic lurasidone was approved in Japan in 2020 as an effective treatment for depressive symptoms in bipolar disorder^14)^. In addition, aripiprazole for long-acting injection has a new indication for the prevention of recurrent manic episodes in bipolar I disorder^15)^.

## Lithium

Lithium is the basic drug used in the pharmacotherapy of bipolar disorder and is listed as the first-line drug in the treatment guidelines of many countries and it is included in the WHO Model List of Essential Medicines (https://list.essentialmeds.org/). In Japan, lithium carbonate is used. Lithium is effective in improving manic and depressive states, preventing recurrence of manic and depressive states, and preventing suicide. However, lithium is associated with many side effects, and because of the proximity of the safe and toxic concentrations, regular measurement of blood levels is necessary. Blood levels should be measured at the beginning of treatment and once every two to three months after stabilization. The effective blood level is between 0.4 and 1.2 mM, and toxicity is more likely to occur when the level exceeds 1.5 mM.

Side effects such as diarrhea, anorexia, thirst, polydipsia, and polyuria are seen when lithium is started. Hand tremor may persist even in the effective concentration range. In the case of intoxication, various symptoms appear, such as cerebellar ataxia, gait disturbance, consciousness disturbance, and vomiting. Since hypothyroidism is often observed, TSH, free T3, and free T4 should be checked regularly. Even if hypothyroidism is observed, lithium treatment can be continued by taking thyroid hormone.

Since concomitant use of non-steroidal anti-inflammatory drugs may increase lithium blood levels, attention should also be paid to concomitant medications. Lithium toxicity is more likely to occur during dehydration. Long-term use of the drug may cause renal dysfunction due to interstitial nephritis, etc. Therefore, a regular check of renal function is necessary. Calcium levels should also be measured periodically while taking lithium, as lithium may cause hyperparathyroidism.

Lithium is contraindicated in pregnancy in Japan due to the increased risk of cardiovascular malformations in the fetus when taken during pregnancy, but it has been reported that there is no significant increase in risk when the dose is 600 mg or less per day^16)^, and revisions to the drug information are expected.

Various other side effects have also been reported, but due to the limited availability of medications for the treatment of bipolar disorder, we should not give up easily when side effects occur and consider measures to minimize them.

## Anticonvulsants

Among the anticonvulsants used as mood stabilizers, lamotrigine alone is indicated for the maintenance treatment of bipolar disorder in Japan. It is effective in preventing relapses and recurrences of all episodes, but the main effect of lamotrigine is to prevent depressive episodes.

Valproic acid has been shown to be effective in manic states. Clinical trials of its prophylactic effect have not been successful, and it does not show a significant prophylactic effect against placebo, and its prophylactic effect is reported to be inferior to that of lithium, but it is also used as a prophylactic drug in clinical practice because meta-analyses suggest its efficacy.

Carbamazepine was found to have an effect on manic states in Japan. Some small trials are suggesting a prophylactic effect, but the evidence is insufficient.

Of these antiepileptic drugs, valproic acid and carbamazepine are indicated by insurance for the measurement of blood levels under the name of manic-depressive illness.

## Antipsychotics

For the manic state of bipolar I disorder, many atypical and typical antipsychotics have been effective, as well as lithium, valproate, and carbamazepine.

For the depressive state of bipolar disorder, three atypical antipsychotics, quetiapine, olanzapine, and lurasidone, have been shown to be effective and are indicated in Japan.

Although not covered by insurance, prophylactic effects have been shown for olanzapine, aripiprazole, and quetiapine. The prophylactic effect of quetiapine is mainly for the prevention of depression. In addition, sustained injection of aripiprazole has been shown to prevent relapses and recurrences of bipolar disorder and was covered by insurance in 2020. The preventive effect of aripiprazole is only for the prevention of manic states.

## Psychotherapy

Psychotherapy alone is not effective in treating bipolar disorder. However, psycho-education is essential to help the patient understand the illness and to encourage and assist acceptance of the illness by paying attention to the patient’s mental reactions to it^17)^. Psycho-education aims to help patients understand the nature of the disease, the effects and side effects of medications, as well as to help them and their families understand and share what the first signs of relapse are. Therefore, it is important to discuss and confirm the initial signs of relapse with the family and share them with the patient and the family. It is also meaningful to predict in advance the stressors that are likely to trigger relapse and to learn how to cope with them.

It is also important to keep a regular life in the treatment of bipolar disorder, as even one-night sleep deprivation can trigger a manic state. Interpersonal social rhythm therapy is reported to be effective in preventing episodes in bipolar disorder. However, it is difficult to receive this treatment in Japan, and usually, the essence of social rhythm therapy is included in psychoeducation; e.g., avoiding staying up all night, get some sunlight in the morning, light exercise such as taking a walk in the morning, and avoiding excessive social stimulation when the mood is unstable.

Cognitive-behavioral therapy (CBT), which is widely used for depression, has also been reported to be effective in preventing the recurrence of bipolar disorder. CBT is available at least in urban areas in Japan.

## Etiology

Bipolar disorder has a higher concordance rate in monozygotic twins than in dizygotic twins, so there is no doubt that genomic factors are involved^18)^. On the other hand, even in monozygotic twins with almost the same genome, not all of them develop the disease, and it is considered that non-genetic factors such as environmental factors are also involved^19)^. Perinatal factors, such as perinatal complications, influenza infection during pregnancy, and smoking of mothers, are reported as environmental factors that pose a risk of bipolar disorder. Early developmental adversity has been reported to have a negative impact on symptoms and course. Stress is said to trigger the onset and recurrence, but it cannot be said to be the cause.

## Genome research

As mentioned above, genome-wide association studies (GWAS) have been conducted because of the involvement of genetic factors in bipolar disorder^20)^. Sixty-four relevant genomic loci were identified in a GWAS of 41,917 bipolar patients^21)^. Among these, *FADS2* (Fatty Acid Desaturase 2) and *FADS1*, which were first found in a GWAS of approximately 3,000 Japanese patients^22)^, are genes for enzymes involved in the metabolism of unsaturated fatty acids, and their reduced activity has been associated with bipolar disorder. In addition to the calcium (Ca^2+^) channel gene *CACNA1C*, which was one of the first genes found in the GWAS for bipolar disorder, a new association was found with another Ca^2+^ channel gene, *CACNB2*. The risk genes for bipolar disorder included many genes involved in synapses, Ca^2+^ signaling, and neurogenesis. In addition, many genes encoding target proteins of antipsychotics, antiepileptic drugs, and other drugs were included.

We performed exome analysis in 354 trio families (patients and their parents) of bipolar disorder and analyzed de novo mutations, i.e., mutations that were not present in the parents^23)^. As a result, we found that de novo mutations were more common in genes with few loss-of-function mutations in the general population. The loss-of-function de novo mutations were more common in genes such as presynaptic active zones and ion channels. In addition, somatic mosaic mutations were frequently found in genes that cause neurodevelopmental disorders, indicating that the presence of genes that cause neurodevelopmental disorders in a somatic mosaic state may be a risk factor for bipolar disorder.

## Neurobiological studies

Although various pathological hypotheses for bipolar disorder have been proposed, the calcium hypothesis, which has been previously reported to be associated with high intracellular Ca^2+^ levels and has been found to be relevant in recent genomic studies, is the most likely. However, since intracellular Ca^2+^ affects many cells, it is difficult to understand the pathogenesis of bipolar disorder by itself^11)^.

We have been focusing on the relationship between mitochondria and bipolar disorder based on the results of magnetic resonance spectroscopy in bipolar disorder patients showing impaired energy metabolism and high levels of mitochondrial DNA (mtDNA) mutations in postmortem brains of patients. It has been reported that patients with mitochondrial diseases have a high rate of bipolar disorder (around 20%)^24)^.

Therefore, we created transgenic mice that express a mutant of polymerase gamma (*Polg*, mtDNA synthetase), one of the genes responsible for mitochondrial disease, only in the brain. The mice exhibited recurrent hypoactive episodes that lasted about two weeks^25)^. This condition occurred on average once every six months, and detailed behavioral analysis showed that they met the diagnostic criteria for a depressive episode (loss of interest, sleep disturbance, increased appetite, slow movements, fatigability, and impaired social behavior). During lithium treatment, these episodes became less frequent, and the patients showed increased corticosteroids during the episodes. In addition, tricyclic antidepressants caused manic-like behavioral changes.

To clarify the brain region responsible for this hypoactivity, we searched for brain regions with high accumulation of mtDNA mutations and found the highest accumulation in the paraventricular thalamic nucleus (PVT). When we manipulated the neuronal circuits of the PVT in mice, similar hypo-active episodes appeared, suggesting that the depression in the model mice was caused by dysfunction of the PVT.

The PVT receives strong projections from serotonergic neurons and is a brain region with high serotonin concentration. It is unique in that it projects to both the amygdala, which is involved in the fear, negative emotion and the nucleus accumbens, which is involved in the reward, positive emotion^26)^.

In another mouse model of mitochondrial disease (*Ant1* mutant mice), serotonin neurons showed hyperexcitability^27)^. Hyperexcitability is also suggested in a study of neurons derived from induced pluripotent cells of patients with bipolar disorder^28)^. Neurons in the PVT may also be hyperexcitable in the mutant POLG mice described above. If overexcitability of the serotonin neuron-PVT system is involved in the pathogenesis of bipolar disorder, we may be able to understand the pathogenesis of this disorder, which presents depressive and manic states in which both negative and positive emotions are extremely enhanced.

These findings suggest the entire picture of bipolar disorder. Genomic factors result in impaired intracellular Ca^2+^ regulation, which leads to hyperexcitability of emotion-related neural circuits, resulting in impaired emotion/cognition balance^11)^.

The gene that regulates excitability in the PVT is a T-type Ca^2+^ channel, and valproic acid is known to be its inhibitor.

Among the serotonin receptors, serotonin 5-HT7 receptors have a characteristic distribution of being abundant in the PVT^29)^, and it is noteworthy that lurasidone is a blocker of serotonin 5-HT7 receptors.

## Mechanism of action of therapeutic agents

The most common theory of the mechanism of action of lithium is inhibition of inositol monophosphatase (IMPase). Inhibition of IMPase causes intracellular depletion of inositol, resulting in diminished agonist-stimulated inositol phospholipid metabolism, which results in attenuation of intracellular Ca^2+^ mobilization. It is also suggested that GSK-3*β* inhibition also plays a role in lithium’s action.

In a study that screened compounds that inhibit IMPase, a drug called ebselen was found, and recently it was suggested that ebselen may be effective in the manic state of bipolar disorder^30)^, which may support the theory that the mechanism of action of lithium is via IMPase inhibition.

On the other hand, for antiepileptic drugs, many attempts have been made to search for common effects with lithium, and intriguing results were obtained. However, no consensus has been reached. Recent genomic studies have found associations with genes involved in neuronal excitability, including Ca^2+^ channels, and iPS cells derived from bipolar disorder patients^28)^, as well as our studies in animal models, have indicated hyperexcitability of neurons. It makes sense to think that these antiepileptic drugs are acting by modulating neuronal excitability, similarly to their action to epilepsy.

Most antipsychotics antagonize the dopamine D2 receptor, which is thought to be the major mode of action for schizophrenia. Most antipsychotics are also effective in manic states, and their effects on manic states are also thought to be mediated by dopamine D2 receptor antagonism. Atypical antipsychotics have fewer extrapyramidal symptoms through a variety of mechanisms, but it is still unclear why some atypical antipsychotics are effective for depressive states in bipolar disorder and others are not. Olanzapine, quetiapine, and lurasidone, which are atypical antipsychotics effective for depression in bipolar disorder, all are antagonists of serotonin receptors, which may be involved in their mechanism of action.

Cognitive-behavioral therapy is a treatment that normalizes the cognitive patterns that predispose to depression, such as overgeneralization and all- or-nothing thinking. These characteristic cognitive patterns are the very characteristic information processing of emotions, and cognitive behavioral therapy may normalize the emotion/cognition balance. Currently, lithium, antiepileptic drugs, atypical antipsychotics, and cognitive-behavioral therapy are often used in combination in clinical situations, and they are thought to act at different points in the pathophysiological pathway of bipolar disorder.

## Funding

No funding to declare.

## Author contributions

TK. wrote the manuscript.

## Conflicts of interest statement

Dr. Kato reports grants and personal fees from Japan Agency for Medical Research and Development (AMED), grants and personal fees from Ministry of Education, Culture, Sports, Science and Technology (MEXT)/Japan Society for the Promotion of Science (JSPS), during the conduct of the study; personal fees from Kyowa Hakko Kirin Co., Ltd., personal fees from Eli Lilly Japan K.K., grants and personal fees from Otsuka Pharmaceutical Co., Ltd., personal fees from GlaxoSmithKline K.K., personal fees from Taisho Pharma Co., Ltd., grants and personal fees from Dainippon Sumitomo Pharma Co., Ltd., personal fees from Meiji Seika Pharma Co., Ltd., personal fees from Pfizer Japan Inc., personal fees from Mochida Pharmaceutical Co., Ltd., grants and personal fees from Shionogi & Co., Ltd., personal fees from Janssen Pharmaceutical K.K., personal fees from Janssen Asia Pacific, personal fees from Yoshitomiyakuhin, personal fees from Astellas Pharma Inc., personal fees from Nippon Boehringer Ingelheim Co. Ltd., personal fees from MSD K.K., personal fees from Kyowa Pharmaceutical Industry Co., Ltd., grants and personal fees from Takeda Pharmaceutical Co., Ltd., personal fees from Taisho Pharmaceutical Co., Ltd., personal fees from Taisho Toyama Pharmaceutical Co., Ltd., grants and personal fees from Eisai Co., Ltd., grants and personal fees from Mitsubishi Tanabe Pharma Corporation, grants from Teijin Pharma, outside the submitted work.
